# The price continuity, return and volatility spillover effects of regular and after-hours trading

**DOI:** 10.1371/journal.pone.0299207

**Published:** 2024-03-11

**Authors:** Chien-Liang Chiu, Ting-Huan Chang, I-Fan Hsiao, De-Shin Chiou

**Affiliations:** 1 Department of Banking and Finance, Tamkang University, New Taipei City, Taiwan; 2 Department of Finance, Chien Hsin University of Science and Technology, Taoyuan, Taiwan; 3 Department of Banking and Finance, Chinese Culture University, Taipei City, Taiwan; Universiti Malaysia Sabah, MALAYSIA

## Abstract

This study employs a bivariate EGARCH model to examine the Taiwan Futures Exchange’s regular and after-hours trading, focusing on the critical aspects of spillover and expiration effects, as well as volatility clustering and asymmetry. The objective of this study is to observe the impact on the trading sessions in Taiwan by the influences of the European and American markets, focusing on the essential roles of the price discovery function and risk disclosure effectiveness of the regular hours trading. This research is imperative considering the increasing interconnectedness of global financial markets and the need for comprehensive risk assessment for investment strategies. It also examines the hedging behavior of after-hours traders, thereby aiming to contribute to pre-investment analysis by future investors. This examination is vital for understanding the dynamics of after-hours trading and its influence on market stability. Results indicate price continuity between both trading sessions, with regular trading often determining after-hours price ranges. Consequently, after-hours price changes can inform regular trading decisions. This finding highlights the importance of after-hours trading for shaping market expectations. Significant profit potential exists in after-hours trading open interest, which serves speculative and hedging purposes. While regular trading volatility influences after-hours trading, the reverse is not true. This suggests Taiwan market information poses a higher risk impact than European and American market data, emphasizing the unique position of the Taiwan market in the global financial ecosystem. After-hours trading volatility reflects the absorption of international market information and plays a crucial role in advance revelation of risks. This underscores the importance of after-hours trading in global risk management and strategy formulation.

## Introduction

The futures market plays a crucial role in financial ecosystems, performing key functions such as speculation, hedging, and price discovery. A well-established futures market is vital for the overall health of the securities market. In this context, the Taiwan Futures Exchange (TAIFEX) introduced the first futures contract (code: TX) based on the Taiwan Capitalization Weighted Stock Index (referred to as the Taiwan Weighted Index) on July 21, 1998. This significant move was followed by the launch of the first options contract (code: TXO) based on the same index on December 24, 2001, thus providing investors with diverse derivatives for hedging or speculative purposes.

However, the regular trading session for TX and TXO, which extends from 8:45 a.m. to 1:45 p.m., was only marginally longer (15 minutes) than the trading hours of the underlying asset. This limited time frame meant that any unsettled positions had to bear overnight risk. Consequently, investors often turned to the Morgan Taiwan Index Futures (code: MTW) traded on the Singapore Exchange Derivatives Trading Limited (SGX) for risk mitigation. This preference was especially pronounced during global events like the 9/11 terrorist attacks, the 3/19 shooting incident, and the 3/11 Fukushima nuclear disaster, which all occurred outside of Taiwan’s regular trading hours, thus triggering systemic risk.

Recognizing the need to mitigate such risks, TAIFEX initiated after-hours trading on May 15, 2017, extending trading hours from 3 p.m. after the current regular session to 5 a.m. the following day. This extension to nearly round-the-clock trading (from 5 hours to 19 hours) significantly strengthened integration with European and American markets. In an era marked by the rapidly increasing efficiency of global financial market risk transmission, this change provided investors with additional channels for hedging and speculation, thereby enhancing the international competitiveness of Taiwan’s futures market.

Futures contracts typically employ a regular trading settlement and mark-to-market system to calculate maintenance margin and mitigate default risk. To prevent potential manipulation of end-of-regular trading prices, which could influence regular trading settlements and the next day’s opening auction reference price, the Taiwan Futures Exchange calculates the settlement price using the volume-weighted average price of all trades conducted within the final minute before closing. Notably, TX and TXO are traded in both regular and after-hours sessions on the same exchange but are considered separate markets with distinct opening, highest, lowest, and closing prices. The settlement price is calculated only during the regular trading session. On the temporal axis, the after-hours session precedes the regular session; the after-hours session that follows the close of regular session t is considered as part of regular session t+1. Unsettled positions at the close of the after-hours session are merged into the regular session t+1 and marked to market using the settlement price from regular session t. Consequently, the opening price for regular session t+1 is based on the settlement price from regular session t, not the closing price of the after-hours session.

Despite the late introduction of after-hours trading in Taiwan compared to other markets, studies focusing on the relationship between returns and volatility in regular and after-hours trading are sparse. This study aims to explore the association between returns and risk in regular and after-hours trading of TX, particularly examining the price discovery function from after-hours to regular trading. Employing a bivariate EGARCH (Exponential General Autoregressive Conditional Heteroskedastic) model, the study investigates the spillover and expiration effects of returns and volatility between these trading sessions, highlighting their inherent volatility clustering and asymmetry. The EGARCH model’s primary advantage is its ability to ensure positive variance without constraining the signs of estimated parameters, and its capacity to differentiate the impacts of positive and negative news.

This study’s primary contributions are twofold: Firstly, it addresses the lack of research on the interconnected effects of Taiwan’s futures after-hours trading on the regular session. Given Taiwan’s status as an island economy with limited resources and high trade-dependency, its futures market is extremely sensitive to international political and economic events and the dynamics of global capital markets. The trading hours of Taiwan’s futures night session align with the daytime hours of the European and American stock markets, offering a unique perspective on the interconnectedness of global financial activities. Secondly, the study reveals that the regular trading session generally experiences lower return volatility compared to the after-hours session. Despite this, the opening prices of both sessions do not show significant differences in average spread. The after-hours session demonstrates efficient price discovery in response to European, American, and post-market Taiwanese information. This indicates that the implementation of after-hours trading effectively reflects overnight information, thereby reducing its impact on the subsequent regular trading session. Returns during after-hours trading tend to be positively influenced by the regular session’s returns, suggesting a continuity in price trends across sessions. However, while regular session volatility often carries over into the after-hours session, this does not consistently extend into the next day’s trading. This pattern implies that risks originating from the Taiwanese market have a more pronounced impact than those from European or American markets. The after-hours session proactively assimilates risks related to information from these foreign markets and Taiwan’s post-market disclosures. These findings provide valuable insights for investors in strategizing across different market sessions.

The paper is structured into six sections: The first section introduces the study; the second section reviews the advancing literature; the third details the bivariate EGARCH model setup; the fourth discusses data sources and statistical test results, providing insights into the characteristics of TX data in regular and after-hours trading, as well as the causal relationship between after-hours and subsequent regular trading prices; the fifth section presents empirical results, elaborating on the spillover and expiration effects of returns and volatility between the two trading sessions, along with their inherent clustering and asymmetry in volatility; the final section concludes the study with a summary of its findings and implications.

## Literature review

The interplay between regular trading hours and after-hours trading has captured substantial academic attention, especially considering the profound impact these hours have on stock returns [1]. Our study is inspired by the notably sparse in-depth research focusing on the Taiwanese market. Through a comprehensive review, we integrate insights from a multitude of studies, providing a unified perspective on the interplay of trading periods and their subsequent impacts on market behavior.

### Price discovery and market efficiency

Price discovery functions predominantly in after-hours trading, showing stronger responses to news released outside standard trading times [2–5]. Pre-market sessions, while efficient in their own right, show a different pattern of price discovery, heavily influenced by trading volumes [6, 7]. The extension of futures trading time, particularly in after-hours, bolsters the efficiency of the ETF market, displaying a more profound reflection of undisclosed information [8–10]. However, the efficiency of price discovery in after-hours trading is relatively low compared to regular trading session [11].

### Market response to earnings announcements and news

Public information, such as earnings announcements, insider trading activities, and index adjustments, is strategically released after market closure, impacting after-hours trading like TX trading. This practice enhances the function of price discovery in after-hours sessions, preparing the market for the upcoming regular trading period [10].

### Trading volumes, liquidity, and short selling

Trading volumes are integral to liquidity and price influence, with regular sessions characterized by larger volumes and robust price discovery functions [12]. In contrast, after-hours trading exhibits limited liquidity but heightened information asymmetry, suggesting a preference for trend-following strategies in short-selling over contrarian approaches [13–15].

### Volatility predictability and trading hours

After-hours trading metrics, including returns and volatility estimates, have shown a predictive capacity for volatility during regular sessions [16, 17]. Nevertheless, this predictive power diminishes when compared to forecasts for pre-market trading volatility [18, 19]. Extended trading hours in after-hours sessions result in greater price fluctuations, indicating a more comprehensive reflection of undisclosed information [8].

### Institutional and investor behavior

The behavior of institutional and foreign investors significantly influences intraday market dynamics, particularly during after-hours trading. This includes considerations for mutual fund trading strategies in response to shareholder meeting outcomes [20, 21].

### Non-trading effects and hedging

Research into the non-trading effects of futures, using TX as a sample, reveals higher holding returns during non-trading periods. Limited liquidity in after-hours sessions leads to increased hedging costs, thereby failing to mitigate the non-trading impact [22].

### International perspectives

The unique dynamics of specific exchanges and commodities, in relation to trading hours, are explored, providing international perspectives on these market behaviors [1].

Our literature review elucidates the complex interplay between regular and after-hours trading across U.S. futures and global markets. It highlights the nuances of market behavior, trading strategies, and potential regulatory implications, offering a comprehensive understanding of financial market behaviors in various trading periods.

## Methodology

This study employs the bivariate EGARCH model to investigate the correlation between regular and after-hours trading of TX, estimating the spillover effects of returns and volatility between regular and after-hours trading, as well as the asymmetry effects of information impacts on volatility. Another pivotal development in financial econometrics—ARCH (Autoregressive Conditional Heteroskedasticity) was introduced, specifically designed to tackle the complexities of time-varying conditional heteroskedasticity [23]. Building on this foundational work, the GARCH (Generalized ARCH) model was proposed. This innovation, a broadened form of the ARCH model, was adept at encapsulating the volatility clustering phenomenon, a common occurrence stemming from conditional heteroskedasticity within the residuals of mean equations [24]. Despite its advanced methodology, the GARCH model exhibited limitations, particularly its inability to discern the asymmetric volatility often triggered by disparate positive and negative information shocks.

To address this shortcoming, the univariate EGARCH (Exponential GARCH) model was further introduced, enabling the analysis of volatility asymmetries, a feature not accommodated by the standard GARCH model [25]. Extending this theoretical framework, and venturing into the multivariate realm, a more comprehensive EGARCH model was formulated, which could interpret interactions and conditions more complex than its univariate predecessor [26].

The returns of regular and after-hours trading of TX are calculated using the logarithmic difference transformation formula [27], as suggested.


$$R_{1t}=ln(S_{t}/P_{t})\times 100$$ (1)

$$R_{2t}=ln(P_{t}/S_{t-1})\times 100$$ (2)

**Fig 1:**
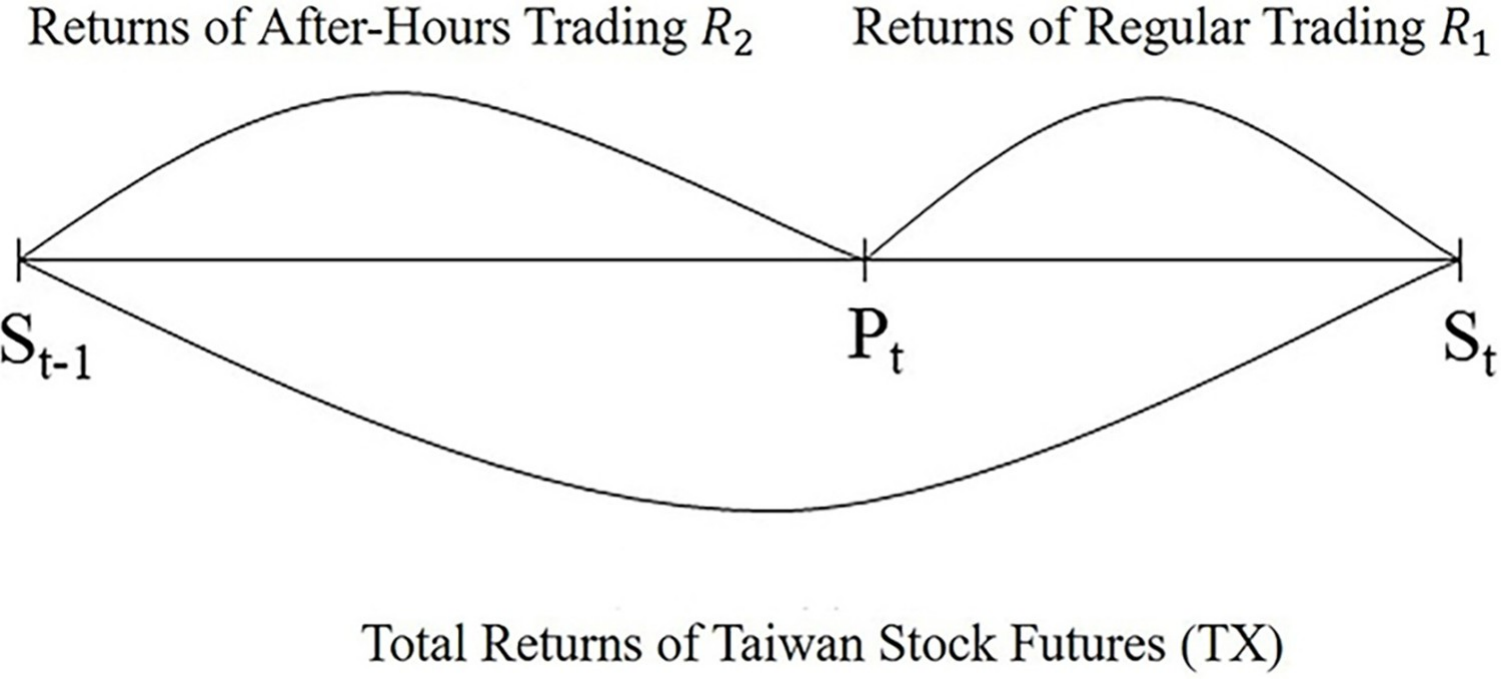
The returns definition for regular and after-hours trading sessions (covering all information on Taiwan Stock Futures returns).

In the Eqs (1) and (2), *R* represents returns, and the subscripts 1 and 2 denote regular and after-hours trading, respectively. Therefore, *R*_1*t*_ and *R*_2*t*_ represent the regular and after-hours trading returns in the *t*-th period. *S* represents the regular trading settlement price published by the Taiwan Futures Exchange, where the final settlement price of *S* is determined by the simple arithmetic average of the Taiwan Weighted Index provided by the Taiwan Stock Exchange within the 30 minutes prior to the close of trading on the same day as the regular trading. *P* represents the closing price of the after-hours trading session. The regular trading return *R*_1*t*_ of TX in Eq (1) is calculated using the logarithmic difference between the *t*-th period of after-hours closing price *P* and the *t*-th period regular trading settlement price *S*. The settlement price *S*_*t*_ on the contract expiration date is the final settlement price announced by the Taiwan Futures Exchange. The *t*-th period regular trading settlement price *S* serves as the opening auction reference for the *t*+1 period of the after-hours trading session, so the after-hours trading return *R*_2*t*_ in Eq (2) is calculated using the logarithmic difference between the *t*-th period closing price *P* and the *t*−1 period settlement price *S*, as shown in Fig 1. The returns definition for regular and after-hours trading sessions covers all information on TX returns.

The returns and volatility estimate of regular and after-hours trading for TX are included in the mean equation of the bivariate EGARCH model. The optimal lag order for both returns series is selected using the SBC (Schwartz’s Bayesian Criterion) and is found to be one lag period. Therefore, the model is set up as follows

$$R_{1t}=\beta_{10}+\beta_{11}R_{1t-1}+\beta_{12}R_{2t-1}+\beta_{13}V_{1t}+\varepsilon_{1t}$$ (3)

$$R_{2t}=\beta_{20}+\beta_{21}R_{1t-1}+\beta_{22}R_{2t-1}+\beta_{23}V_{2t}+\varepsilon_{2t}$$

$$\varepsilon_{it}|\Omega_{t-q}∼N(0,\sigma_{it}^{2})\;i=1,2$$

In the above equations, *β*_10_ and *β*_20_ are the intercept terms. *β*_11_ and *β*_22_ represent the autoregressive parameter estimates for the first lag of regular and after-hours trading returns, respectively. *β*_12_ and *β*_21_ are the parameter estimates for the spillover effects from the *t*−1 period of after-hours and regular trading returns to the ones in the *t*-1 period, respectively. The variables *V*_1_ and *V*_2_ are the log differences of regular and after-hours trading volumes, representing the trading volume volatility, which are beneficial for observing the implied risk aversion behavior triggered by the returns impact. Therefore, *β*_13_ and *β*_23_ represent the parameter estimates for the impact of trading volume volatility on their respective current returns of the *t*−1 period. *ε*_*it*_ is the random error term, and Ω_*t*−*q*_ represents the information set up to the *t*−1 period. It is assumed that *ε*_*it*_ follows a normal distribution with a mean of zero and a variance of $\sigma_{it}^{2}$.

The conditional heteroskedasticity variance equation for the bivariate EGARCH model is set up as follows

$$\sigma_{1t}^{2}=\exp\left(\alpha_{10}+\gamma_{11}f_{1}(Z_{1t-1})+\gamma_{12}f_{2}(Z_{2t-1})+\delta_{1}\ln\left(\sigma_{1t-1}^{2}/m_{1t-1}^{2\omega_{1}}\right)\right)$$

$$\sigma_{2t}^{2}=\exp\left(\alpha_{20}+\gamma_{21}f_{1}(Z_{1t-1})+\gamma_{22}f_{2}(Z_{2t-1})+\delta_{2}\ln\left(\sigma_{2t-1}^{2}/m_{2t-1}^{2\omega_{2}}\right)\right)$$ (4)

$$\sigma_{12t}=\rho_{12}\sigma_{1t}\sigma_{2t}$$

$$f_{i}(Z_{it-1})=(\left|Z_{it-1}|-E|Z_{it-1}\right|)+\theta_{i}Z_{it-1}\;i=1,2$$ (5)

$$\frac{\partial f_{i}(Z_{it})}{\partial Z_{it}}=1+\theta_{i}\;if\;Z_{it-1}\geq 0$$ (6)

$$\frac{\partial f_{i}(Z_{it})}{\partial Z_{it}}=-1+\theta_{i}\;if\;Z_{it-1}<0$$ (7)

In the above Eqs (4) to (7), *α*_10_ and *α*_20_ are the intercept terms for volatility, measuring the volatility of regular and overnight markets at 1 lag, as well as the spillover effects from the *t*−1 period volatility of regular and after-hours trading to the ones in the *t*−1 period, respectively. The volatility is derived from |*Z*_*it*−1_|−*E*|*Z*_*it*−1_||Z_(it-1), where *Z*_*it*_ = *ε*_*it*_/*σ*_*it*_. *Z*_*it*−1_ represents the standardized residuals of regular and after-hours trading returns in the *t*−1 period, and *γ*_11_, *γ*_12_, *γ*_21_, and *γ*_22_ are their parameter estimates. *δ*_1_ and *δ*_2_ are used to measure the volatility clustering phenomenon. As *m*_1_ and *m*_2_ are the number of days until the expiration date (the third Wednesday of each month) of the TX futures contracts, *ω*_1_ and *ω*_2_ are used to measure the expiration effect of volatility. If *ω* is not significant, there is no expiration effect; if it is significantly positive, the conditional heteroskedasticity variance decreases as the expiration date approaches; if it is significantly negative, the conditional heteroskedasticity variance increases as the expiration date approaches. *ρ*_12_ is the constant, representing the correlation coefficient between the two conditional heteroskedasticity sequences.

Additionally, in Eq (5), *θ*_*i*_ is used to measure the asymmetric volatility phenomenon. When *Z*_*it*−1_≥0, the slope is 1+*θ*_*i*_; when *Z*_*it*−1_<0 the slope is −1+*θ*_*i*_. A negative value for *θ*_*i*_ causes the degree of volatility to increase; in contrast, a positive value for *θ*_*i*_ causes the degree of volatility to decrease, set in Eqs (6) and (7).

Assuming a normal distribution, the log-likelihood function for the bivariate EGARCH model is as follows

$$L(\Phi )=\frac{1}{2}(NT)ln(2\pi )-\frac{1}{2}\sum \left(ln|\psi_{t}|+\varepsilon_{t}^{\prime }\psi_{t}^{-1}\varepsilon_{t}\right)$$ (8)

In the Eq (8), N is the number of mean equations, T is the number of observations, Φ is the estimated coefficient matrix of 23×1, *ε*′_*t*_ = (*ε*_1*t*_, *ε*_2*t*_) is the 1×2 residual vector matrix for period *t*, and *ψ*_*t*_ is the 2×2 time-varying conditional variance and covariance matrix. When the model has a high degree of nonlinearity, it is necessary to use an iterative algorithm to obtain the maximum log-likelihood function value. In this study, the BHHH algorithm is used to estimate the coefficient values of the parameters listed in the model [28].

To test the goodness of fit, the Ljung-Box Q test is used for residual autocorrelation; the ARCH test for residual heteroskedasticity. To determine if the model successfully captures the asymmetric phenomenon of volatility, the Engle and Ng asymmetry test is used [29]. The Sign Bias Test (SBT) is defined as $Z_{it}^{2}\equiv {\left(\varepsilon_{it}/\sigma_{it}\right)}^{2}=a+bS_{it}+e_{it}$, where *S*_*it*_ is a dummy variable that takes the value of 1 when *ε*_*it*_≤0, and 0 otherwise. The Negative Size Bias Test (NSBT) is defined as $Z_{it}^{2}\equiv {\left(\varepsilon_{it}/\sigma_{it}\right)}^{2}=a+bS_{it}\varepsilon_{it-1}+e_{it}$, and the Positive Size Bias Test (PSBT) is defined as $Z_{it}^{2}\equiv {\left(\varepsilon_{it}/\sigma_{it}\right)}^{2}=a+b(1-S_{it})\varepsilon_{it-1}+e_{it}$. The Joint Test (JT) is defined as $Z_{it}^{2}\equiv {\left(\varepsilon_{it}/\sigma_{it}\right)}^{2}=a+b_{1}S_{it}+b_{2}S_{it}\varepsilon_{it-1}+b_{3}(1-S_{it})\varepsilon_{it-1}+e_{it}$, which follows a chi-square distribution with 3 degrees of freedom.

## Data sources and statistical test results

The regular and after-hours trading prices, settlement prices, and trading volumes of TX are all obtained from the Taiwan Futures Exchange. The sample period is from May 18, 2017, to May 18, 2022; namely, covering the daily data of the TX nearby contract following the establishment of the after-hours trading system (expires in June, 2017) to the 60th (expires in May, 2022), and the dataset includes 1,220 sample observations. The starting point of May 18, 2017 was chosen for a specific reason. This date follows closely after the Taiwan Futures Exchange introduced the after-hours trading system on May 15, 2017. However, we avoided starting immediately after the implementation due to May 17, 2017, being a settlement day (the third Wednesday of the month). To ensure the integrity and clarity of our data analysis, the commencement of the complete nearest month contract was selected, which begins from May 18, 2017. Since the sample period begins with the first complete near-month contract month following the official implementation of after-hours trading, the examination process of the methodology allows for the omission of robustness test issues.

During the initial implementation of after-hours session trading for Taiwan Stock Index Futures, if there was a make-up workday on a Saturday, regular trading session trading would proceed as usual, while after-hours session trading would be suspended. Consequently, regular trading data for June 5, 2017, October 2, 2017, April 2, 2018, and December 24, 2018, were removed. In addition, after-hours session trading on July 11, 2018, was suspended due to Typhoon Maria, and the corresponding regular trading data was also excluded. Since January 1, 2019, both stock and futures markets have been closed on make-up workdays on Saturdays, resulting in no more instances of incomplete data.

Table 1 shows the ADF (Dickey and Fuller), PP (Phillips and Perron) and KPSS (Kwiatkowski, Phillips, Schmidt and Shin.) unit root tests. The regular and after-hours returns of the TX are significant, while the KPSSunit root test is not, indicating that both return series are stationary [30–32].

**Table 1:** The unit-root test results for the Taiwan Stock Futures trading returns in regular and after-hours sessions rate of return.

Method	Model	Regular		Lags	After-Hours		Lags
ADF	Constant	-17.3480	**	3	-10.2138	**	10
	Constant and Linear Trend	-17.3424	**	3	-10.2124	**	10
	No Constant and Linear Trend	-17.2086	**	3	-9.9319	**	10
PP	Constant	-36.5908	**	4	-38.1432	**	11
	Constant and Linear Trend	-36.5780	**	4	-38.1300	**	11
KPSS	Constant	0.1082		4	0.1118		11
	Constant and Linear Trend	0.0912		4	0.0980		11

Note

** indicate the significance level of the test statistic at 1%.

Table 2 presents the basic descriptive statistics of the regular and after-hours trading returns of the TX. It can be noticed that the regular trading session has a negative mean return but is not significantly different from 0, while the after-hours session has a positive mean return that is significantly different from 0. However, the standard deviation of the regular trading session is smaller than that of the after-hours session, indicating that the regular trading session has smaller return fluctuations and lower risk. Such results suggest that the after-hours session reflects the overnight risk information of European and American stock markets in advance, reducing the volatility of regular trading session returns. The Jarque-Bera normality test shows a significant rejection of the normal distribution assumption, and the kurtosis coefficient is also significant, indicating that both return series exhibit leptokurtic and fat-tailed distribution characteristics. The Pearson correlation coefficient provides a significantly low positive correlation between regular and after-hours trading returns, indicating the worthiness to further investigate the correlation between regular and after-hours sessions of TX trading regarding returns and volatility.

**Table 2:** Descriptive statistics of Taiwan Stock Futures trading returns in regular and after-hours sessions.

	Regular Session		After-Hours Session	
Mean	-0.0053		0.0451	*
S.D.	0.0093		0.6167	
Min.	-8.0473		-5.3521	
Mas.	6.0880		2.6831	
Skewness	-1.2031	**	-1.3785	**
Kurtosis	12.2554	**	13.2296	**
JB	7922.6926	**	5701.1393	**
Correlation Coefficient	-0.0370	**		
Q(5)	48.7004	**	29.0918	**
Q^2^(10)	350.9141	**	504.6375	**
ARCH(5)	169.2748	**	300.8988	**
ARCH(10)	174.6420	**	329.3397	**

Note

1. *and ** indicate the significance level of the test statistic at 5% and 1%, respectively.

2. JB represents the Jarque-Bera test for normality.

3. This study defines a correlation coefficient between 0 and 0.3 as a low degree of correlation, between 0.3 and 0.7 as a moderate degree of correlation, and between 0.7 and 1 as a high degree of correlation.

4. Q(5) and Q^2^(10) represent the Ljung-Box Q test statistics for the return sequence and the squared return sequence at lags 5 and 10, respectively.

5. ARCH(5) and ARCH(10) represent the chi-squared test statistics for conditional heteroskedasticity of the squared return sequence at lags 5 and 10, respectively

Additionally, the Ljung-Box Q(5) and Q^2^(10) statistics for both regular and after-hours trading in their return series and the squared return series are significant, indicating that both return series exhibit linear cross-time dependence and serial correlation. The ARCH(5) and ARCH(10) tests for the ARCH effect are also significant, indicating that both return series have time-varying conditional heteroskedasticity. Therefore, GARCH-type models are appropriate for such estimation. Consequently, this study employs the bivariate EGARCH model to capture the clustering and asymmetry of volatility heteroskedasticity.

Although the after-hours trading of TX takes place on *t*+1 day, any open positions from this session are merged into the regular trading session on the *t* day, and the profit and loss are calculated based on the settlement price of regular trading of the *t* day, therefore making decisions on whether to hold the position have become an important issue for after-hours investors. In Table 3, based on the after-hours session as the benchmark, the average price differences are compared. The average price difference between the regular trading session’s opening price and the after-hours session’s closing price is not significant at 1.99 points, indicating that there is a price continuity phenomenon between the regular trading session’s opening price and the after-hours session’s closing price. The average price difference between the regular trading session’s settlement price and the after-hours session’s closing price is significant at -20.75 points, which may be due to the impact of information during the regular trading session. In addition, the average price difference between the regular trading session’s highest price and the after-hours session’s highest price is significant at 20.82 points, while the average price difference between the regular trading session’s lowest price and the after-hours session’s lowest price is significant at -13.80 points. Therefore, there is a profit margin for the price difference in the regular trading session for both long and short open positions in the after-hours session.

**Table 3:** Average price difference between regular trading session and after-hours session.

	Regular Opening VS After-Hours Closing		Regular Highest VS After-Hours Highest		Regular Lowest VS After-Hours Lowest		Regular Settlement VS After-Hours Closing	
Average Price Difference	1.99		20.82	**	-13.80	**	-20.75	*
Correlation Coefficient	0.9870	**	0.9596	**	0.9489	**	0.5459	**

Note

1. *and ** indicate the significance level of the test statistic at 5% and 1%, respectively.

2. This study defines a correlation coefficient between 0 and 0.3 as a low degree of correlation, between 0.3 and 0.7 as a moderate degree of correlation, and between 0.7 and 1 as a high degree of correlation.

Table 3 also presents the correlation coefficients, which indicate a significantly strong positive correlation between the regular trading session’s opening price and the after-hours session’s closing price, the regular trading session’s highest price and the after-hours session’s highest price, and the regular trading session’s lowest price and the after-hours session’s lowest price. The regular trading session’s settlement price and the after-hours session’s closing price exhibit a significantly moderate positive correlation. The above results show that there is price continuity between the regular and after-hours sessions, and it is common for the after-hours session’s highest and lowest prices to be tested. Hence, the price changes in the after-hours session can serve as a reference for the price changes in the regular trading session. Moreover, the range between the regular trading session’s highest and lowest prices is larger than that of the after-hours session, with significant price differences. Therefore, investors with open positions in the after-hours session can profit from the price difference by closing their positions in the regular trading session, providing both hedging and speculative functions during the regular trading session.

## Empirical results

This study investigates the mutual influences between regular and after-hours sessions of TX trading based on returns and volatility, as well as their volatility clustering and asymmetry. Table 4 presents the results of the estimation of the bivariate EGARCH model for the TX regular and after-hours trading sessions. For model goodness of fit diagnostics, Ljung-Box Q(5) and Q^2^(20) test statistics are insignificant, indicating that there is no linear cross-temporal dependence in the residual sequences of the two returns; the ARCH effects tests for ARCH(5) and ARCH(10) are also insignificant, suggesting that there is no conditional heteroskedasticity in both return residual sequences; therefore, proper goodness of fit can be confirmed. In addition to the insignificant joint test results of SBT, NSBT, PSBT, and JT, the model is believed to successfully captures the asymmetry effects of both volatility sequences.

**Table 4:** EGARCH model estimation result.

Parameters	Regular		Parameters	After-hours	
Panel A: Mean equation					
*β*_10_	0.0002(0.0005)		*β*_20_	0.0006(0.0002)	**
*β*_11_	-0.0876(0.0311)	**	*β*_21_	0.0599(0.0158)	**
*β*_12_	0.3653(0.0667)	**	*β*_22_	-0.0306(0.0335)	
*β*_13_	0.0066(0.0006)	**	*β*_23_	-0.0019(0.0003)	**
*β*_14_	-0.0040(10^−2^) (0.0038(10^−2^))		*β*_24_	-0.0001(10^−2^) (0.0018(10^−2^))	
Panel B: Variance equation					
*α*_10_	-0.0978(0.4921)		*α*_20_	-0.8681(0.4219)	*
*γ*_11_	0.0453(0.0414)		*γ*_21_	0.2838(0.0441)	**
*γ*_12_	0.0354(0.0464)		*γ*_22_	0.4648(0.0471)	**
*δ*_1_	0.9683(0.0519)	**	*δ*_2_	0.9326(0.0391)	**
*ω*_1_	0.0596(0.0227)	**	*ω*_2_	-0.0297(0.0233)	
*θ*_1_	-5.9759(5.5296)		*θ*_2_	-0.1007(0.0704)	
*ρ*_12_	0.2146(0.0546)	**			
Maximum likelihood estimates 8738.3910					
Panel C: Model Diagnosis					
Q(5)	4.4796		Q(5)	4.2692	
Q^2^(10)	2.7505		Q^2^(10)	11.3406	
ARCH(5)	0.9766		ARCH(5)	2.9857	
ARCH(10)	2.7733		ARCH(10)	11.5668	
SBT	0.1031		SBT	0.1338	
NSBT	-0.5346		NSBT	7.9923	
PSBT	-3.5615		PSBT	-3.9521	
JT	0.6800		JT	4.3481	

Note

1. *and ** indicate the significance level of the test statistic at 5% and 1%, respectively.

2. JB represents the Jarque-Bera test for normality.

3. This study defines a correlation coefficient between 0 and 0.3 as a low degree of correlation, between 0.3 and 0.7 as a moderate degree of correlation, and between 0.7 and 1 as a high degree of correlation.

4. Q(5) and Q^2^(10) represent the Ljung-Box Q test statistics for the return sequence and the squared return sequence at lags 5 and 10, respectively.

5. ARCH(5) and ARCH(10) represent the chi-squared test statistics for conditional heteroskedasticity of the squared return sequence at lags 5 and 10, respectively.

Table 4 presents the parameter estimates for the mean equation. The estimated coefficients for the self-influence parameters *β*_11_ and *β*_22_, which affect the returns of the TX trading sessions, are significantly negative for the regular trading session, but insignificant for the after-hours session. This indicates that the regular session trading returns are negatively affected by previous returns, while the after-hours session returns are not affected by previous returns. The cross-influence parameters *β*_12_ and *β*_21_ have estimated coefficients that are significantly negative for the regular trading session and significantly positive for the after-hours trading session, respectively. This implies that the regular session trading returns are negatively affected by the previous returns of the after-hours session, suggesting that the after-hours session efficiently absorbs information from European and American markets and Taiwan’s after-hours information disclosure, resulting in efficient price discovery. Therefore, the implementation of the after-hours session trading system can alleviate the potential impact on the regular trading session. On the other hand, the after-hours session trading returns are positively affected by the previous trading returns of the regular session, indicating that the regular trading session generates price continuity expectations in the same direction for the after-hours trading session, with investors generally adopting trend-following strategies and not easily changing their interpretation of information in the regular trading session. The estimated coefficients for the parameters *β*_13_ and *β*_23_, which capture the effects from trading volume and volatility to the regular and after-hours trading, are both significantly negative, indicating that both the regular and after-hours session trading returns are negatively affected by the current trading volume volatility. This is evident in the hedging behavior of investors who actively short futures in anticipation of a decline in spot prices.

Table 4 presents the parameter estimates for the conditional heteroskedastic variance equation. The estimated coefficients for the self-influence parameters *β*_11_ and *β*_22_, which affect the volatility of the TX trading sessions, are insignificant for the regular trading session and significantly positive for the after-hours trading session. This suggests that the volatility of the regular trading session is not affected by the volatility from the previous session; whilst, the volatility of the after-hours trading session is significantly and positively affected by the previous session. The cross-influence estimated coefficients, *γ*_12_ and *γ*_21_, are insignificant for the regular trading session and significantly positive for the after-hours trading session, indicating that the regular trading session volatility is not affected by the prior after-hours session trading volatility; however, the after-hours session trading volatility is significantly and positively affected by the previous regular trading session volatility. Such result suggests that the regular trading session volatility lasts until the following after-hours trading session, but the after-hours session volatility does not remain afterwards. Since the regular session trades while European and American markets are closed, and the after-hours session trades while those two markets are operating, the after-hours trading session is more influenced by them. This implies that the information of risk generated locally is more concerning for investors compared to ones generated from European and American markets, and the after-hours trading session functions well on absorbing information of foreign risk and local information disclosure, as volatility has reflected it in advance.

The conditional heteroskedastic variance parameters *δ*_1_ and *δ*_2_ have estimated coefficients that are close to 1 and significant for both volatility sequences, indicating that both the regular and after-hours trading sessions of TX markets exhibit volatility clustering; namely, large volatility is followed by large volatility; small volatility is followed by small volatility. The volatility asymmetry parameters *θ*_1_ and *θ*_2_ have insignificant estimation results, indicating that there is no volatility asymmetry phenomenon; for bad news, the impacts are no greater than good news based on similar volatility levels observed in both the regular and after-hours trading sessions. The volatility correlation coefficient parameter *ρ*_12_ has an estimated coefficient that is significantly 0.2146, indicating a low positive correlation between the regular and after-hours trading session volatilities.

Moreover, the estimated coefficients of expiration effect parameters *β*_14_ and *β*_14_ for the regular and after-hours session trading returns are both insignificant, suggesting that the regular and after-hours session trading returns are not affected by the expiration effect. The estimated coefficients of expiration effect parameters *ω*_1_ and *ω*_2_ for the regular and after-hours session volatilities are significantly positive for the former session and are insignificant for the latter ones, indicating that the regular trading session volatility exhibits an expiration effect. Namely, volatility increases as the expiration date approaches, but such an effect does not exist in the after-hours trading session.

**Fig 2:**
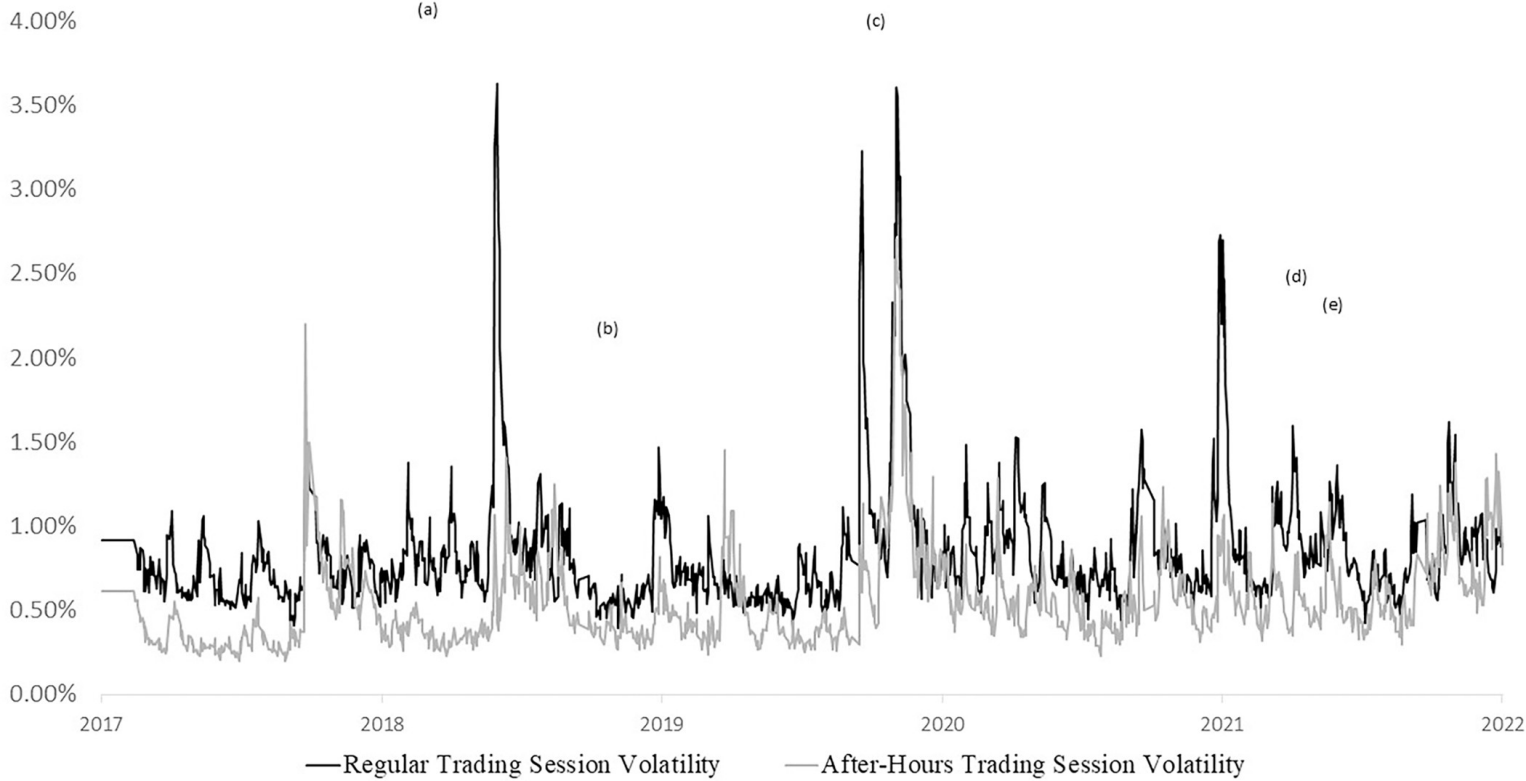
Volatility in regular and after-hours trading session.

Fig 2 shows the dynamic volatility of the regular and after-hours sessions of TX trading, revealing that the regular trading session volatility is generally larger than the after-hours session volatility, and they tend to follow the same pattern. The noticeable bad events affecting the risks of the regular and after-hours sessions are: (a) the “Futures Massacre” on February 6, 2018, which happened on February 6th, 2018 in Taiwan’s futures market. At 8:45 am, the Taiwan Index Futures opened at 10,643 points, but dropped around 284 points, about 2.6%. Five minutes later, at 8:50 am, six out-of-the-money call and put options were "instantly" pushed up to the limit price (including the February 9500 put option, which was almost at the limit price). At 8:51 am, five out-of-the-money call and put options were pushed up to the limit price. However, due to the abnormal market prices, the risk indicators of option margins were severely distorted (because abnormal prices were used for calculation), and as a result, futures traders began to aggressively liquidate positions, causing a massacre in the option market. Many out-of-the-money call options (which were expected to decline) rose to their limit prices; (b) the U.S. Department of Commerce’s ruling on October 3, 2018, which accused that China and Italy were dumping steel, leading to the continuous expansion of the U.S.-China trade war; (c) the global outbreak of the COVID-19 pandemic; (d) the U.S. Federal Reserve has announced the policy to end quantitative easing and reduce balance sheet by interest rate hikes in March 2021. During these events, the impact of systemic risk had been reflected in both regular and after-hours trading sessions, where their volatilities significantly increased. Finally, (e) the outage incident of the Hsinta Power Plant in the Taiwan on May 13, 2021, which occurred on May 13th, 2021. The first outage was caused by a ground fault in the busbar of a substation due to an incorrect isolation switch operation during equipment acceptance. This failure caused a sudden drop in voltage, resulting in the tripping of four coal-fired generating units at the Hsinta Power Plant, causing a loss of about 2.2 million kilowatts of power supply and a sudden drop in frequency, which triggered the automatic protection mechanism of the power grid and caused power outages for some customers. It could not immediately restore power in a short period of time to maintain the safety and stability of the power system.

## Conclusion

This study employs the bivariate EGARCH model to estimate the Taiwan Stock Index Futures (TX) from May 18, 2017, to May 18, 2022, covering a complete 60 TX nearby contracts since the implementation of the after-hours session trading. This comprehensive time frame encompasses significant market dynamics, offering a robust analysis of the spillover effects on TX trading returns and volatilities between regular and after-hours trading sessions. Additionally, the study delves into the exploration of the expiration effects, the volatility clustering, and asymmetry among the two distinct trading periods.

The empirical results of this study reveal a notable contrast in volatility between the two trading sessions. Specifically, the regular trading session returns exhibit lower volatility compared to the after-hours session. This finding is further supported by the average price difference observed between the opening price in the regular trading session and the closing price in the after-hours trading session, indicating a presence of a price continuity phenomenon. Intriguingly, the regular trading session often tests the highest and lowest prices set in the after-hours trading session, suggesting that the price changes in the after-hours session can serve as a reliable reference for anticipating price movements in the subsequent regular session trading. Moreover, the analysis reveals that the highest price in the regular trading session is significantly higher than that in the after-hours trading, and vice versa for the lowest prices. Such results indicate a wider price range in the regular trading session than in the after-hours trading session. Therefore, it is inferred that regardless of being long or short, open positions in the after-hours trading session serve both hedging and speculative functions, thus presenting arbitrage opportunities during the regular trading session.

The empirical results from the bivariate EGARCH model demonstrate a dynamic interplay between the two sessions. The regular trading session returns are negatively affected by the previous after-hours session returns, implying that the after-hours session efficiently incorporates foreign market information from Europe, America, and after-hours market information in Taiwan itself. This efficient assimilation of information allows the after-hours session trading system to adequately reflect these inputs, thereby mitigating the impact on the following regular trading session. Conversely, the after-hours session trading returns are positively influenced by the previous regular session trading returns, indicating the existence of price continuity between the two sessions. This finding is pivotal as it suggests that for investors in the after-hours session, it would be rational to adopt a trend-following strategy, refraining from hastily altering their interpretation of regular trading session information. A notable phenomenon observed in both the regular and after-hours trading sessions is that increased trading volume is typically accompanied by a decrease in prices. This trend highlights investors’ proactive short-selling of futures as a strategic response to confront risk. Additionally, the analysis reveals that no expiration effect can be discerned in the trading returns of both sessions, providing insights into market behavior around contract expirations.

In terms of volatility patterns, the study finds that volatility in the TX regular trading session is not significantly influenced by prior volatility. However, the volatility in the after-hours trading session is significantly and positively affected by prior volatility. While volatility in the regular trading session persists into the after-hours trading session, the reverse is not observed, indicating a unidirectional impact. This suggests that the Taiwanese market is more sensitive to information-induced risks generated locally compared to those from European and American markets. Volatility in the after-hours trading session effectively reflects and absorbs risk information from these international markets and post-market information disclosure in Taiwan. Volatility patterns in both regular and after-hours trading sessions exhibit clustering, though neither show asymmetry. An expiration effect can be observed in the regular trading session, as increased volatility is displayed as the expiration date approaches, while such an effect cannot be observed in the after-hours trading session. Moreover, when systemic risk events occurred, volatilities in both regular and after-hours trading sessions simultaneously reflected and increased, underlining the interconnectedness of the two sessions in response to broader market dynamics.

Since May 15, 2017, the Taiwan Futures Exchange (TAIFEX) has implemented an after-hours session trading system following the close of the regular trading session. This innovative development provides investors with a convenient post-market hedging channel, effectively reducing institutional friction in cross-market trading. The after-hours session trading reacts promptly to information from European and American markets and Taiwan’s post-market information. Consequently, its influence on the continuous pricing and price discovery function of the regular trading session should be taken into account for informed investment decisions.

Furthermore, TAIFEX has made various detailed trading data available, enhancing the research scope of the futures market and trading strategies. Building upon this, future studies are encouraged to incorporate intra-regular trading data and compare retail and institutional investors’ decision-making behaviors. Additionally, the hedging effectiveness of regular and after-hours trading sessions may be deeply examined. Ultimately, this study aims to contribute significantly to a better understanding of the characteristics of Taiwan’s futures market and to suggest improvements to the trading system.

Additionally, given that the after-hours trading session of TX overlaps with the regular trading hours of European and American stock markets, the after-hours TX trading is initially influenced by the spot and futures markets in these regions. Therefore, the correlation between these markets and the TX after-hours trading session warrants more in-depth exploration in future research endeavors.
